# Decreased iKIR-HLA C Pair Confers Worse Clinical Outcomes for Patients With Myeloid Disease Receiving Antithymocyte Globulin-Based Haploidentical Hematopoietic Stem Cell Transplantation

**DOI:** 10.3389/fimmu.2020.614488

**Published:** 2021-02-04

**Authors:** Yanmin Zhao, Fei Gao, Yibo Wu, Jimin Shi, Yi Luo, Yamin Tan, Jian Yu, Xiaoyu Lai, Mingming Zhang, Wei Zhang, He Huang

**Affiliations:** ^1^ Bone Marrow Transplantation Center, the First Affiliated Hospital, School of Medicine, Zhejiang University, Hangzhou, China; ^2^ Institute of Hematology, Zhejiang University, Hangzhou, China; ^3^ Zhejiang Engineering Laboratory for Stem Cell and Immunotherapy, Hangzhou, China; ^4^ Zhejiang Blood Center, Hangzhou, China

**Keywords:** KIR, hematopoietic stem cell transplantation, iKIR-HLA model, relapse, survival

## Abstract

Hematopoietic stem cell transplantation (HSCT) is a curative therapy for patients with malignant hematologic diseases. Killer immunoglobin-like receptor (KIR) expressed by NK cells is closely associated with the transplant outcomes, and it has been widely explored and debated for a few decades. Recently published studies have revealed that inhibitory KIRs (iKIRs) are educated by their cognate human lymphocyte antigen (HLA) ligands, and that decreased iKIR-HLA pairs post-transplantation may indicate a reduced NK cell function and impaired control of the primary disease. However, this theory still needs to be validated by additional clinical studies. Here we conducted a retrospective analysis of 246 patients who received haploidentical (haplo)-HSCT at our treatment center between January 2015 and June 2018. Our data suggests that decreased iKIR-HLA C pair post-HSCT correlated with a significantly higher risk of relapse [hazard risk (HR) = 2.95, p = 0.019] and reduced overall survival (OS) (HR = 3.74, p = 0.001) and disease-free survival (DFS) (HR = 4.05, p = 0.0004) in patients with myeloid disease. In conclusion, decreased iKIR-HLA C pair should be avoided during anti-thymocyte globulin (ATG)-based haplo-HSCT, especially for patients with myeloid disease.

## Introduction

Natural killer (NK) cells act as the first line of defense in the immune system. They can rapidly recognize autologous cells and eliminate non-self-components without prior antigen presentation (1, 2). Multiple receptors expressed on NK cells have been implicated in the regulation of their function, with a particular focus on the activities of killer immunoglobin-like receptors (KIRs).

It is well accepted that KIR genes and receptors can be divided into inhibitory and activating functions based on their diverse activities (3). Inhibitory KIRs (iKIRs) bind human lymphocyte antigen (HLA) class I molecules in a specific manner, KIR2DL1 recognizes HLA-C2 group allies, KIR2DL2 and KIR2DL3 recognize HLA-C1 group allies, KIR3DL1 recognizes HLA-Bw4 group allies, and KIR3DL2 recognizes HLA-A3/A11 allies. Activating KIRs (aKIRs) such as KIR2DS1, KIR2DS2, and KIR2DS4 recognize HLA C2, HLA C1, and HLA A11, respectively, but the ligands of the remaining KIRs remain largely unknown. Based on their chromosomal locations, KIR genes can be further identified as centromeric (cen) or telomeric (tel) genes. In addition, KIR genotype AA is made up of only one aKIR gene: KIR2DS4, while KIR genotype B/x is made up of a number of more variable aKIR genes (4).

Normally, autoimmune activation is inhibited because autologous cells express at least one inhibitory HLA ligand; however, tumor transformed cells downregulate HLA expression and/or upregulate activating signals that may trigger NK cell activation (5, 6). Following allogeneic hematopoietic stem cell transplantation (allo-HSCT), donor-derived NK cells may be activated as the recipients may not express the same inhibitory HLA ligands as the donor, preventing their association with the donor iKIRs. This has led to widespread speculation that NK cell alloreactivity in graft *versus* host (GVH) direction may provide additional benefits to tumor-killing strategies.

The Perugia group first established the KIR ligand-ligand model (also known as the KIR ligand model) based on HLA phenotype differences between donors and recipients. In this model, they assumed that donor-derived NK cells might kill recipient cells because the HLA ligands presented by the donor might be absent in the recipient. When they evaluated T cell-depleted (TCD) transplants without post-transplant immunosuppression, they were able to show that KIR ligand mismatch between donor-recipient pairs provided some protective effect against relapse, especially in patients with acute myeloid leukemia (AML) (7). Further development of KIR-typing technology allowed researchers to develop the receptor-ligand model (also known as the missing ligand model), which was used to evaluate the compatibilities between donor iKIRs and recipient HLA ligands. Results using this model suggested that the receptor-ligand model was a more accurate predictor for relapse risk than the KIR ligand model in leukemia patients (8). Additionally, Cooley et al. reported that KIR B/x donors significantly improved the relapse-free survival (RFS) rates for recipients with AML when compared to donors with a KIR AA genotype, suggesting that aKIRs may play a critical role in reducing relapse (9). Following these observations, numerous clinical studies have explored the impact of KIR on transplant outcomes. However, a large variability was found in these results and several factors may be responsible for these discrepancies, including disease type, transplant regimen, donor-recipient relationship, graft source and graft composition, etc (10–12).

In the last few decades, our understanding of NK cell reconstitution and KIR education has evolved a great deal. Pioneer studies in this field have found that reconstituted NK cells are highly immature and exhibit compromised cytotoxicity against leukemia cells in the early phases following transplantation. Afterward, these NK cells gradually acquire receptors and KIR reconstitution can take between several months and even years (13, 14). Importantly, a variety of data has suggested that NK cells acquire specific functionality only after engagement between the iKIRs and their cognate ligands. However, NK cells expressing iKIRs without cognate ligands (non-self KIR) are hyporesponsive and referred to “uneducated cells” (15, 16). Further, the education process mediated by cognate ligands is not restricted to autologous NK cells, but has also been demonstrated in donor-derived reconstituted NK cells after HSCT (17–19).

Recently, the Nowak team proposed the iKIR-HLA model to explore the optimal donor. Since the HLA environment may be altered after transplantation (from donor to recipient), the variations in iKIR-HLA pairs could be divided into three groups (decreased group: cognate iKIR-HLA pairs present in donor but absent in recipient; unchanged group: cognate iKIR-HLA pairs present both in donor and recipient; increased group: cognate iKIR-HLA pairs present in recipient but absent in donor). Consistent results from their studies showed that decreased iKIR-HLA pairs post transplantation correlated with a higher risk of relapse and inferior overall survival (OS), indicating that poor NK cell education resulted in weaker graft *versus* leukemia (GVL) effects (20–22). To further investigate the effects of these KIR interactions on transplant outcomes, we designed a retrospective study to evaluate a cohort of 246 patients, and evaluated our clinical outcomes using the iKIR-HLA model, the receptor-ligand model and KIR gene content.

## Methods

### Patients

This retrospective study was comprised of 246 patients with hematological malignancies. All transplants were performed between January 2015 and June 2018 and all methodologies applied during this study were consistent with the Declaration of Helsinki. The protocol was approved by the Ethics Review Committee of the First Affiliated Hospital of Zhejiang University and informed consent was obtained from each patient before transplantation. The authors had full access to the data and assume responsibility for its authenticity.

### KIR and HLA Typing

Peripheral blood mononuclear cells were collected from recipients and their donors prior to transplantation and used for HLA and KIR testing. Alleles in the HLA-A, -B, and -C loci were determined using high-resolution HLA typing and KIR gene analysis was performed using the PCR-SSO method (KIR SSO Genotyping Test; OneLamda, Canoga Park, CA, USA).

### Transplant Protocol

Most patients were subjected to a myeloablative conditioning (MAC) regimen that included administration of cytarabine (4 g/m^2^/d IV on days −10 to −9), busulfan (Bu) (3.2 mg/kg/d IV on days −8 to −6), cyclophosphamide (Cy) (1.8 g/m^2^/d IV on days −5 to −4), methyl-N-(2-chloroethyl)-N-cyclohexyl-N-nitrosourea (Me-CCNU) (250 mg/m^2^ orally on day −3), and antithymocyte globulin Fresenius [ATG-F; 2.5 mg/(kg d) IV on days −5 to −2] or ATG Genzyme [ATG-G; 1.5 mg/(kg d) IV on days −5 to −2]. The other patients were subjected to reduced-intensity conditioning (RIC) that consisted of exposure to fludarabine 30 mg/m^2^/d IV between days −10 and −5, Bu 3.2 mg/kg/d IV between days −6 and −5, and ATG-F 5 mg/(kg d) IV between days −4 and −1 or ATG-G 2.5 mg/(kg d) IV between days −4 and −1. All patients received G-CSF mobilized peripheral blood stem cells and no graft was subjected to *ex vivo* T-cell depletion. Graft *versus* host disease (GVHD) prophylaxis consisted of cyclosporin A (CsA) or Tacrolimus (Tac), with methotrexate (MTX) and low-dose mycophenolate mofetil (MMF) (23, 24).

### Definitions

Relapse was defined as disease reoccurrence in bone marrow and/or extramedullary sites. Non-relapse mortality (NRM) was defined as death from any cause apart from relapse. Overall survival (OS) was defined as the time from transplant until death or last follow up, and disease-free survival (DFS) was defined as survival without relapse. Patients were classified as low/intermediate risk or high/very high risk based on the refinement of the disease risk index (DRI) (25). Diagnosis of acute and chronic GVHD (aGVHD and cGVHD) was made using established criteria (26, 27). The viral loads for Epstein-Barr virus (EBV) and cytomegalovirus (CMV) were monitored weekly for the first 3 months after transplantation, biweekly from the fourth to the sixth month post-transplant, and monthly from the seventh to the twelfth month post-transplant. Viremia was defined as a viral load in excess of 5 × 10^2^ copies/ml.

### Statistical Analysis

All clinical data were analyzed using SPSS 22.0 (IBM, Armonk, NY, USA) and R project 3.6.1 software (http://www.r-project.org). The clinical features for the samples were presented as median or percentage values. OS and DFS were calculated using the Kaplan–Meier method and compared using the log-rank test. The cumulative incidences of EBV viremia, CMV viremia, aGVHD, cGVHD, relapse, and NRM were estimated *via* the competing-risks model and compared using the Gray test. All variables with a p-value of <0.10 in the univariate analysis were then included in the multivariate analysis. Results were considered statistically significant when p < 0.05.

## Results

### Characteristics of Patients and Donors

The clinical features of these 246 donor-patient pairs are summarized in **Table 1**. In this retrospective study, 142 (57.7%) patients with myeloid disease and 104 (42.3%) patients with lymphoid disease received haplo-HSCT at our center. Disease types included acute myeloid leukemia (AML, n = 115), myelodysplastic syndrome (MDS, n = 22), myeloproliferative neoplasm (MPN, n = 5), acute lymphoblastic leukemia (ALL, n = 93), and lymphoma (n = 11). The median age of the patients and donors in these groups were 30 years (range, 9–50 years) and 35 years (range, 11–59 years), respectively. The median mononuclear (MNC) cell and CD34^+^ cell counts in the grafts were 15.34 × 10^8^/kg (range, 2.97–59.80 × 10^8^/kg) and 6.30 × 10^6^/kg (range, 0.27–34.37 × 10^6^/kg), respectively. A total of 233 (94.7%) patients received the MAC regimen and 13 (5.3%) received the RIC regimen. ATG-F was used in 205 (83.3%) patients while the other 41 (16.7%) received ATG-G as part of their conditioning regimen. One hundred eighty-one (73.5%) patients received haplo-HSCT during their first remission (CR1); 73 (29.7%) patients were defined as high or very high risk based on the refinement of DRI (49 in the myeloid cohort and 24 in the lymphoid cohort, 34.5 *vs* 23.1%, p = 0.053). Most patients expressed HLA C1C1 or HLA C1C2 and only 4.5% presented with a HLA C2C2 ligand.

**Table 1:** Clinical features of patients, donors, and transplants.

Variables	All patients (246)	Myeloid cohort (142)	Lymphoid cohort (104)
Median patient age (years)	30 (9–60)	33 (9–60)	24 (13–56)
Median donor age (years)	35 (11–59)	32 (11–55)	38 (13–59)
Median MNC cells (×10 E^8^/kg)	15.34 (2.97–59.80)	14.36 (2.97–59.80)	15.61 (5.80–46.14)
Median CD34^+^ cells (×10 E^6^/kg)	6.30 (0.27–34.37)	6.06 (0.27–34.37)	7.03 (1.77–22.87)
Median follow up time (years)	3.0 (0.1–5.5)	3.0 (0.2–5.5)	2.9 (0.1–5.5)
Patient sex			
Male Female	136 (55.3) 110 (44.7)	77 (54.2) 65 (45.8)	59 (56.7) 45 (43.3)
Donor/Patient sex combination			
Female/Male Other combinations	44 (17.9) 202 (82.1)	27 (19.0) 115 (81.0)	17 (16.3) 87 (83.7)
ABO blood mismatch			
Identical Mismatch	131 (53.3) 115 (46.7)	73 (51.4) 69 (48.6)	58 (55.8) 46 (44.2)
Diagnosis		/	/
AML MDS MPN ALL Lymphoma	115 (46.7) 22 (8.9) 5 (2.0) 93 (37.8) 11 (4.5)		
Disease status at HSCT			
CR1 >CR1	181 (73.5) 65 (26.4)	99 (69.7) 43 (30.3)	82 (78.9) 22 (21.2)
Disease risk index			
Low/Int risk High/Very high risk	173 (70.3) 73 (29.7)	93 (65.5) 49 (34.5)	80 (76.9) 24 (23.1)
Conditioning regimen			
MAC RIC	233 (94.7) 13 (5.3)	133 (93.7) 9 (6.3)	100 (96.2) 4 (3.8)
ATG			
ATG-F ATG-G	205 (83.3) 41 (16.7)	117 (82.4) 25 (17.6)	88 (84.6) 16 (15.4)
HLA ligands of patients			
A3/A11^+^ Bw4^+^ C1/C1 C1/C2 C2/C2	115 (46.7) 148 (60.2) 167 (67.9) 68 (27.6) 11 (4.5)	66 (46.5) 90 (63.4) 95 (66.9) 42 (29.6) 5 (3.5)	49 (47.1) 58 (55.8) 72 (69.2) 24 (23.1) 6 (5.8)
Receptor-ligand (R-L) model			
R-L A3/A11 mismatch R-L Bw4 mismatch R-L C mismatch	131 (53.3) 96 (39.0) 176 (71.5)	76 (53.5) 49 (34.5) 98 (69.0)	55 (52.9) 47 (45.2) 78 (75.0)
Donor KIR genotype			
AA B/x BA BB	143 (58.1) 103 (41.9) 76 (30.9) 27 (11.0)	81 (57.0) 61 (43.0) 45 (31.7) 16 (11.3)	62 (59.6) 42 (40.4) 31 (29.8) 11 (10.6)
Donor activating KIR gene			
KIR2DS1^+^ KIR2DS2^+^ KIR2DS3^+^ KIR2DS4^+^ KIR2DS5^+^ KIR3DS1^+^	83 (33.7) 46 (18.7) 41 (16.7) 238 (96.7) 57 (23.2) 85 (34.6)	49 (34.5) 27 (19.0) 24 (16.9) 135 (95.1) 36 (25.4) 51 (35.9)	34 (32.7) 19 (18.3) 17 (16.3) 103 (99.0) 21 (20.2) 34 (32.7)
iKIR-HLA pairs variation			
A3/A11			
Decreased (D) Unchanged (U) Increased (I)	40 (16.2) 166 (67.5) 40 (16.2)	22 (15.5) 98 (69.0) 23 (16.2)	18 (17.3) 68 (65.4) 17 (16.3)
Bw4			
Decreased (D) Unchanged (U) Increased (I)	26 (10.6) 189 (76.8) 31 (12.6)	14 (9.9) 109 (76.8) 19 (13.4)	12 (11.5) 80 (76.9) 12 (11.5)
C			
Decreased (D) Unchanged (U) Increased (I)	43 (17.5) 163 (66.3) 40 (16.3)	20 (14.1) 98 (69.0) 24 (16.9)	23 (22.1) 65 (58.7) 16 (15.4)
EBV viremia	90 (36.6)	41 (28.9)	49 (47.1)
CMV viremia	160 (65.0)	93 (65.5)	67 (64.4)
aGVHD			
Grade 0 Grade I Grade II Grade III Grade IV	92 (37.4) 71 (28.9) 55 (22.4) 12 (4.9) 16 (6.5)	55 (38.7) 44 (31.0) 30 (21.1) 7 (4.9) 6 (4.2)	37 (35.6) 27 (26.0) 25 (24.0) 5 (4.8) 10 (9.6)
cGVHD			
Not included No Mild Moderate Severe	7 (2.8) 139 (56.5) 58 (23.6) 26 (10.6) 16 (6.5)	2 (1.4) 76 (53.5) 39 (27.5) 14 (9.9) 11 (7.7)	5 (4.8) 63 (60.6) 19 (18.3) 12 (11.5) 5 (4.8)
relapse	55 (22.4)	28 (16.9)	27 (26.0)
NRM	14 (5.7)	4 (2.8)	10 (9.6)
OS	185(75.2)	115 (82.4)	70 (67.3)
DFS	177 (72.0)	110 (80.3)	67 (64.4)

AML, acute myeloid leukemia; MDS, myelodysplastic syndrome; MPN, myeloproliferative neoplasm; ALL, acute lymphoblastic leukemia; MNC, mononuclear; CR1, first complete remission; MAC, myeloablative conditioning; RIC, reduced-intensity conditioning; ATG, Antithymocyte Globulin; EBV, Epstein-Barr virus; CMV, cytomegalovirus; aGVHD, acute graft versus host disease; cGVHD, chronic graft versus host disease; NRM, non-relapse mortality; OS, overall survival; DFS, disease-free survival.

Of the 246 donors, 143 (58.1%) were KIR genotype AA, 76 (30.9%) were KIR BA, and 27 (11.0%) were KIR BB. Receptor-ligand (R-L) mismatches at the HLA-A3/A11 locus, HLA-Bw4 locus, and HLA-C locus were identified in 53.3, 39.0, and 71.5% of the donor-recipient pairs, respectively. After transplantation, 40 (16.2%) patients experienced a decrease in their iKIR-HLA A3/A11 pair, 26 (10.6%) exhibited decreased iKIR-HLA Bw4 pair, and 43 (17.5%) had decreased iKIR-HLA C (C1 or C2) pair.

### EBV and CMV Viremia

During the first 180 days after HSCT, 90 (36.6%) patients developed EBV viremia. Disease category (myeloid or lymphoid) (p = 0.001), ATG source (p = 0.0003), and patient sex (p = 0.029) were identified as potent factors influencing EBV viremia (**Table 2**). Multivariate analysis suggested that myeloid disease [hazard risk (HR) = 0.48, p = 0.0005] was a protective factor for EBV viremia, while ATG-G (HR = 2.58, p < 0.0001) and sex (male patients (HR = 1.57, p = 0.042)) were independent risk factors for EBV viremia (**Table 3**). In lymphoid disease, KIR2DS2^+^ donors were found to exhibit a higher incidence of EBV viremia when compared with KIR2DS2^−^ donors (63.2 *vs* 43.5%, p = 0.078), but this was not identified to be an independent effect in the multivariate analysis.

**Table 2:** Univariate analysis of factors that influence transplant outcomes.

Outcome and potent factors, %	All patients	P	Myeloid cohort	P	Lymphoid cohort	P
1. EBV viremia*						
Myeloid *vs* Lymphoid	28.9 *vs* 47.1	**0.001**				
ATG-G *vs* ATG-F	58.6 *vs* 32.2	**0.0003**	48.0 *vs* 24.8	**0.009**	75.0 *vs* 42.1	**0.003**
Male *vs* Female	42.7 *vs* 29.1	**0.029**	35.1 *vs* 21.5	**0.087**	52.5 *vs* 40.0	0.177
KIR2DS2^+^ *vs* KIR2DS2^−^	43.5 *vs* 35.0	**0.199**	25.0 *vs* 29.7	0.643	63.2 *vs* 43.5	**0.078**
2. CMV viremia*						
ATG-G *vs* ATG-F	78.1 *vs* 62.4	**0.003**	72.0 *vs* 64.1	0.199	87.5 *vs* 60.4	**0.001**
KIR2DS1^+^ *vs* KIR2DS1^−^	67.5 *vs* 63.8	0.935	77.6 *vs* 59.1	**0.029**	52.9 *vs* 70.0	**0.030**
KIR2DS3^+^ *vs* KIR2DS3^−^	63.4 *vs* 65.4	0.695	79.2 *vs* 62.7	0.191	41.2 *vs* 69.0	**0.057**
KIR3DS1^+^ *vs* KIR3DS1^−^	67.9 *vs* 63.6	0.997	78.0 *vs* 58.7	**0.041**	52.9 *vs* 70.0	**0.030**
R-L C (mismatch *vs* match)	62.5 *vs* 71.4	**0.079**	63.3 *vs* 70.5	0.267	61.5 *vs* 73.1	0.151
3. Grade II-IV aGVHD*						
High/Very high risk *vs* Low/Int risk	41.1 *vs* 30.6	**0.080**	38.8 *vs* 25.8	**0.068**	45.8 *vs* 36.3	0.454
MAC *vs* RIC	35.2 *vs* 7.7	**0.041**	31.6 *vs* 11.1	0.178	40.0 *vs* 0.0	0.125
R-L C (mismatch *vs* match)	31.25 *vs* 40.0	0.256	29.6 *vs* 31.8	0.853	33.3 *vs* 53.9	**0.095**
4. Moderate to severe cGVHD*						
KIR2DS2^+^ *vs* KIR2DS2^−^	6.5 *vs* 19.2	**0.048**	7.4 *vs* 20.3	0.133	5.3 *vs* 17.7	0.197
KIR2DS3^+^ *vs* KIR2DS3^−^	7.3 *vs* 18.7	**0.083**	12.5 *vs* 19.0	0.487	0.0 *vs* 18.4	**0.057**
5. 3-yr CIR						
Myeloid *vs* Lymphoid	17.3 *vs* 26.2	**0.087**				
High/Very high risk *vs* Low/Int risk	32.9 *vs* 16.1	**0.002**	26.5 *vs* 12.4	**0.031**	45.8 *vs* 20.4	**0.009**
CR1 *vs* >CR1	16.5 *vs* 33.9	**0.002**	13.6 *vs* 25.7	**0.070**	23.5 *vs* 36.4	0.202
iKIR-HLA C variation (D *vs* U+I)	38.1 *vs* 17.5	**0.005**	40.0 *vs* 13.5	**0.004**	35.8 *vs* 23.5	0.317
6. 3-yr NRM						
Myeloid *vs* Lymphoid	2.8 *vs* 9.9	**0.024**				
High/Very high risk *vs* Low/Int risk	1.4 *vs* 7.7	**0.057**	2.0 *vs* 3.2	0.683	0.0 *vs* 12.8	**0.070**
iKIR-HLA C variation (D *vs* U+I)	4.7 *vs* 6.0	0.745	10.0 *vs* 1.6	**0.037**	0.0 *vs* 12.7	**0.078**
KIR2DS3^+^ *vs* KIR2DS3^−^	0.0 *vs* 7.0	**0.086**	0.0 *vs* 3.4	0.362	0.0 *vs* 11.8	0.145
KIR B/x *vs* KIR AA	2.9 *vs* 7.9	0.112	0 *vs* 4.9	**0.079**	7.1 *vs* 11.7	0.497
7. 3-yr OS						
Myeloid *vs* Lymphoid	81.6 *vs* 67.7	**0.016**				
ATG-G *vs* ATG-F	75.5 *vs* 75.8	0.861	92.0 *vs* 79.3	0.147	49.2 *vs* 71.0	**0.041**
High/Very high risk *vs* Low/Int risk	67.0 *vs* 79.3	**0.036**	71.4 *vs* 87.1	**0.029**	58.3 *vs* 70.5	0.202
CR1 *vs* >CR1	79.7 *vs* 64.5	**0.010**	85.8 *vs* 72.0	**0.053**	72.4 *vs* 50.0	**0.025**
iKIR-HLA C variation (D *vs* U+I)	65.1 *vs* 77.9	**0.093**	55.0 *vs* 86.0	**0.0006**	73.9 *vs* 65.9	0.418
8. 3-yr DFS						
Myeloid *vs* Lymphoid	79.9 *vs* 63.9	**0.006**				
ATG-G *vs* ATG-F	73.2 *vs* 73.3	0.835	78.3 *vs* 88.0	0.293	50.0 *vs* 66.5	**0.085**
High/Very high risk *vs* Low/Int risk	65.7 *vs* 76.2	**0.080**	71.4 *vs* 84.4	**0.066**	54.2 *vs* 66.8	0.218
CR1 *vs* >CR1	76.7 *vs* 63.0	**0.024**	83.3 *vs* 72.0	**0.107**	67.7 *vs* 50.0	**0.085**
iKIR-HLA C variation (D *vs* U+I)	57.3 *vs* 76.5	**0.016**	50.0 *vs* 84.9	**0.0001**	64.2 *vs* 63.9	0.813

*Estimations of cumulative incidence are given at 100 days post-HSCT for aGVHD; 180 days post-HSCT for EBV and CMV viremia; 3 years post-HSCT for cGVHD. Potent factors with p < 0.10 were in bold type.

**Table 3:** Multivariate analysis of factors that influence transplant outcomes.

Outcomes and significant factors	All patients		Myeloid cohort		Lymphoid cohort	
	P	HR (95% CI)	P	HR (95% CI)	P	HR (95% CI)
1. EBV viremia*						
ATG-G *vs* ATG-F	**<0.0001**	**2.58 (1.61–4.13)**	**0.007**	**2.51 (1.28–4.93)**	**0.004**	**2.66 (1.37–5.14)**
Male *vs* Female	**0.042**	**1.57 (1.02–2.41)**				
Myeloid *vs* Lymphoid	**0.0005**	**0.48 (0.31–0.72)**				
2. CMV viremia*						
ATG-G *vs* ATG-F	**0.008**	**1.70 (1.15–2.51)**			**0.005**	**1.76 (1.19–2.59)**
3. 3-yr CIR						
CR1 *vs* >CR1	**0.029**	**0.53 (0.30–0.94)**				
iKIR-HLA C variation (D *vs* U+I)	**0.033**	**1.95 (1.06–3.61)**	**0.019**	**2.95 (1.19–7.27)**		
4. 3-yr OS						
Myeloid *vs* Lymphoid	**0.006**	**0.49 (0.29–0.82)**				
CR1 *vs* >CR1	**0.004**	**0.46 (0.27–0.78)**			**0.029**	**0.45 (0.22–0.92)**
iKIR-HLA C variation (D *vs* U+I)			**0.001**	**3.74 (1.66–8.39)**		
5. 3-yr DFS						
Myeloid *vs* lymphoid	**0.003**	**0.47 (0.28–0.77)**				
CR1 *vs* >CR1	**0.009**	**0.51 (0.31–0.84)**			**0.034**	**0.47 (0.24–0.94)**
iKIR-HLA C variation (D *vs* U+I)			**0.0004**	**4.05 (1.87–8.80)**		

*Estimations of cumulative incidence are given at 100 days post-HSCT for aGVHD; 180 days post-HSCT for EBV and CMV viremia. Significant factors with P < 0.05 were in bold type.

The CI for CMV viremia within 180 days of transplant was 65.0% (78.1% in patients treated with ATG-G and 62.4% in patients treated with ATG-F, p = 0.003). Donor-patient pairs with R-L mismatch at HLA-C locus tended to experience a lower CI for CMV viremia than did donor-patient R-L C matched pairs (62.5 *vs* 71.4, p = 0.079). The multivariate analysis revealed that only ATG-G was an independent risk factor for CMV viremia (HR = 1.70, p = 0.008).

### aGVHD and cGVHD

Following transplantation, 83 (33.7%) developed grade II–IV aGVHD (aGVHD^2–4^). As expected, a significant reduction in aGVHD^2–4^ occurrence was found in patients receiving RIC conditioning compared with patients receiving MAC conditioning (7.7 *vs* 35.2%, p = 0.041). Patients with low and intermediate risk also experienced a lower CI of aGVHD^2–4^ (30.6 *vs* 41.1%, p = 0.080). However, none of these factors remained significant in the multivariate analysis. In lymphoid cohort, there was a trend that donor-patient pairs with R-L mismatch on HLA-C locus experienced a lower aGVHD^2–4^ (33.3 *vs* 53.9%, P = 0.095).

Among patients who survived more than 100 days after transplantation, 100 (41.8%) patients developed cGVHD and 42 (17.6%) of them had moderate to severe cGVHD. Univariate analysis identified KIR2DS2 (p = 0.048) and KIR2DS3 (p = 0.083) as two potent protective factors for moderate to severe cGVHD. Nevertheless, no such correlations were found in the multivariate analysis.

### Relapse and NRM

**Figure 1:**
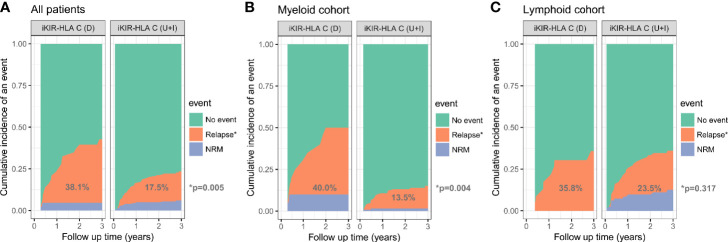
Cumulative incidence of relapse among all patients **(A)**, the myeloid cohort **(B)**, and the lymphoid cohort **(C)**, based on the variation (D, decreased; U, unchanged; I, increased) in iKIR-HLA C pair post-transplantation.

After a median follow up time of 3.0 years (yr) (range, 0.1–5.5 yr), 55 (22.4%) patients experienced relapse. Patients with lymphoid disease experienced a higher 3-yr relapse rate than patients with myeloid disease (26.2 *vs* 17.3%, p = 0.087). The CI for 3-yr relapse was also higher in patients with high/very high-risk disease (32.9 *vs* 16.1%, p = 0.002). Patients who received HSCT at CR1 experienced a lower 3-yr relapse rate than the other group (16.5 *vs* 33.9%, p = 0.002). No significant differences in relapse rate were found using the receptor ligand model and activating KIRs. However, decreased iKIR-HLA C pair were associated with a higher risk for 3-yr relapse (38.1 *vs* 17.5%, p = 0.005). When analyzed separately, the discrepancy in relapse rates was more evident in the myeloid cohort (40.0 *vs* 13.5%, p = 0.004) than in the lymphoid cohort (35.8 *vs* 23.5%, p = 0.317) (**Figure 1**). Multivariate analysis revealed that CR1 (HR = 0.53, P = 0.029) and decreased iKIR-HLA C pair (HR = 1.95, P = 0.033) were independent factors for relapse for the entire cohort, and the adverse effects of decreased iKIR-HLA C pair on the 3-yr relapse rate was more evident in myeloid disease (HR = 2.95, p = 0.019).

A total of 14 (5.7%) patients experienced NRM at a median follow-up time of 0.3 yr (range, 0.1–2.8 yr), 6 (2.4%) patients died of severe GVHD (5 aGVHD and 1 cGVHD), 7 (2.8%) patients died from severe infection (6 pulmonary infections and 1 sepsis), and 1 (0.4%) patient with primary poor graft function (28) died from an intracranial hemorrhage. No variables were found to be significant predictors of NRM.

### OS and DFS

**Figure 2:**
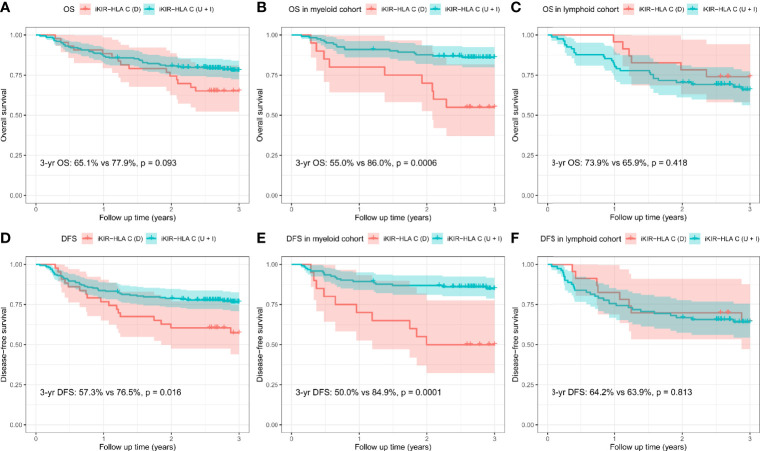
Overall survival (OS) and disease-free survival (DFS) rates for all patients **(A, D)**, the myeloid cohort **(B, E)**, and the lymphoid cohort **(C, F)**, based on the variation (D, decreased; U, unchanged; I, increased) in their iKIR-HLA C pair post-transplantation.

The CI for 3-yr OS was 75.6% for all patients. Disease category (p = 0.016), disease status (p = 0.010), and disease risk index (p = 0.036) were all found to influence 3-yr OS in the univariate analysis. In addition, the 3-yr OS rate in transplants with decreased iKIR-HLA C pair was shown to be 65.1% [95% confidence interval (CI): 52.3–81.0%], which was lower than those with unchanged or increased iKIR-HLA C pair (77.9%, 95% CI: 72.3–83.9%, p = 0.093), and the negative impact of decreased iKIR-HLA C pair was more apparent in the myeloid cohort [55.0% (95% CI: 37.0–81.8%) *vs* 86.0 (95% CI: 80.0–92.4%), p = 0.0006] than in the lymphoid cohort [73.9% (95% CI: 58.0–94.2%) *vs* 65.9% (95% CI: 56.1–77.3%), p = 0.418] (**Figures 2A–C**). In the lymphoid cohort, patients who received ATG-G prior to transplantation experienced a lower 3-yr OS (49.2 *vs* 71.0%, p = 0.041). Multivariate analysis identified myeloid disease (HR = 0.49, p = 0.006) and CR1 (HR = 0.46, p = 0.004) as protective factors for 3-yr OS. CR1 in the lymphoid cohort (HR = 0.45, p = 0.029) remained significant in multivariate analysis, and decreased iKIR-HLA C pair conferred a poorer 3-yr OS in the myeloid cohort (HR = 3.74, p = 0.001).

In addition, dramatically reduced 3-yr DFS was observed when iKIR-HLA C pair was decreased both in the entire cohort [57.3% (95% CI: 44.0–74.6%) *vs* 76.5% (95% CI: 70.8–82.6%), p = 0.016] and the myeloid cohort [50.0% (95% CI: 32.3–77.5%) *vs* 84.9% (95% CI: 78.6–91.6%), p = 0.0001]. For patients with lymphoid disease, variation in iKIR-HLA C pair was not associated with DFS [64.2% (95% CI: 47.0–87.8%) *vs* 63.9% (95% CI: 54.2–75.4%), p = 0.813] (**Figures 2D–F**). In the multivariate analysis, myeloid disease (HR = 0.47, p = 0.003) and CR1 (HR = 0.51, p = 0.009) were shown to be independent factors influencing DFS. A significantly reduced DFS was also observed in myeloid patients with decreased iKIR-HLA C pair (HR = 4.05, p = 0.0004).

## Discussion

There has been a longstanding debate about the impact of KIR alloreactivity on clinical outcomes. It was only recently revealed that reconstituted KIR are educated by HLA ligands and that the loss of the cognate ligands dampens NK cell functions (17–19). This means that searching for donors who exhibit the greatest NK cell function in recipients rather than “match or mismatch” would be a more reliable measure for predicting transplant success.

Previously, Nowak et al. proposed the iKIR-HLA model that could be used to predict transplant outcomes (20–22). Among the multiple interactions between the iKIRs and HLA ligands, we identified that only decreased iKIR-HLA C pair post transplantation was a negative indicator for relapse and survival, especially in patients with myeloid disease. Nevertheless, variations in iKIR-HLA A3/A11 pair and iKIR-HLA Bw4 pair did not influence the treatment outcomes.

It is widely accepted that almost all HLA C molecules are recognized by iKIRs. However, only a minority of HLA B and HLA A epitopes act as KIR ligands (29–31). Similarly, all patients in our cohort expressed at least one HLA C ligand, while the HLA Bw4 and A3/A11 ligands were expressed at a frequency of 60.2 and 46.7%, respectively. This suggests that the HLA C ligands play a dominant role in KIR education (32). Given this, reconstituted NK cells with decreased iKIR-HLA C pair may exhibit impaired anti-tumor effects (18, 19). In addition, the expression levels of HLA A and B ligands on normal cells are more than tenfold higher than that of the HLA C (33), this means that when cancerous cells downregulate HLA class I antigens to escape immune surveillance, the stability of the self-tolerance mediated by iKIR-HLA C interactions is more vulnerable to be broken. In other words, HLA-C may play a major role in missing-self recognition and modulate NK cell activation. Moreover, Pende et al. found that lymphoblastic leukemias express a higher surface density of HLA class I molecules than myeloid leukemias (34). Verheyden et al. went on to test the expression of HLA ligands in normal T cells, AML cells, B-ALL cells, and B-chronic lymphoid leukemic (B-CLL) cells. Interestingly, only HLA C were dramatically downregulated on all types of leukemic cells as compared with their healthy control, with this downregulation being the most apparent in AML cells (35). Makanga et al. demonstrated that CD57^+^ and KIR^+^ NK cells from healthy individuals exhibited the highest degree of cytotoxicity against AML blasts, while ALL targets were less susceptible to KIR^+^ NK subsets compared with NKG2A^+^ NK subsets (36). On the basis of previous studies, we hypothesize that KIR may have a minor impact on the elimination of lymphoblastic leukemias, and patients with myeloid disease are more likely to benefit from well KIR-educated NK cells.

In many European studies, aKIRs, especially KIR2DS1 (37–39) and KIR2DS2 (40, 41), have been shown to be associated with improved survival or reduced relapse. Yet, as reported in several studies from East Asia (42–45), aKIRs were not found to grant any survival advantage or relapse protection to the patients in our cohort. One reason for this may be the genetic differences between these different ethnic groups. Single et al. revealed that almost 46.7% Europeans express the KIR2DS2 gene, and 66.5% present the HLA C2 ligand for KIR2DS1 (46). However, both the KIR2DS2 gene (18.7%) and the HLA C2 ligand (32.1%) were expressed at much lower frequencies in this study. The KIR2DS1 gene frequency in our cohort was also a bit lower than those of the European populations (33.7 *vs* 37.8%). Thus, we speculate that KIR2DS1 may have a reduced chance of activation resulting from the absence of its cognate ligand, and that the beneficial impact of KIR2DS2 on transplant outcomes may be more apparent in a larger cohort of Chinese patients.

Additionally, we could not find evidence of any significant association between receptor ligand mismatch and clinical outcomes. Since mature donor lymphocytes are mostly eliminated following ATG treatment, the transient expression of alloreactive NK cells in the recipients may not be sufficient to influence GVHD (47–49). After which the reconstituted NK cells expressing non-self KIRs may not exhibit enough cytotoxicity to eliminate the remaining leukemic cells (17–19).

In summary, we conclude that when using ATG-based haplo-HSCT, deceased iKIR-HLA C pair should be avoided during donor selection, especially for patients with myeloid disease. The exact role of the aKIRs in the Chinese population still needs to be explored in future studies.

## Data Availability Statement

The original contributions presented in the study are included in the article/supplementary material. Further inquiries can be directed to the corresponding author.

## Ethics Statement

The studies involving human participants were reviewed and approved by the Ethics Review Committee of the First Affiliated Hospital of Zhejiang University. Written informed consent to participate in this study was provided by the participants’ legal guardian/next of kin.

## Author Contributions

HH designed the study and supervised the analyses and manuscript preparation. YZ, FG, and YW collected and analyzed the data, YZ and FG wrote the manuscript. All authors discussed and interpreted the results. All authors contributed to the article and approved the submitted version. YZ and FG contributed equally to this work and should be considered as co-first authors.

## Funding

This work was supported by the National Natural Science Foundation of China (81670148 and 81730008) and Key Project of Science and Technology Department of Zhejiang Province (2019C03016).

## Conflict of Interest

The authors declare that the research was conducted in the absence of any commercial or financial relationships that could be construed as a potential conflict of interest.
